# Comparison of *Plasmodium ovale curtisi* and *Plasmodium ovale wallikeri* infections by a meta-analysis approach

**DOI:** 10.1038/s41598-021-85398-w

**Published:** 2021-03-19

**Authors:** Aongart Mahittikorn, Frederick Ramirez Masangkay, Kwuntida Uthaisar Kotepui, Giovanni De Jesus Milanez, Manas Kotepui

**Affiliations:** 1 Department of Protozoology, Faculty of Tropical Medicine, Mahidol University, Bangkok, Thailand; 2 Department of Medical Technology, Institute of Arts and Sciences, Far Eastern University-Manila, Manila, Philippines; 3 Medical Technology, School of Allied Health Sciences, Walailak University, Tha Sala, Nakhon Si Thammarat, Thailand

**Keywords:** Epidemiology, Infectious diseases

## Abstract

Malaria caused by *Plasmodium ovale* species is considered a neglected tropical disease with limited information about its characteristics. It also remains unclear whether the two distinct species *P. ovale curtisi* and *P. ovale wallikeri* exhibit differences in their prevalence, geographic distribution, clinical characteristics, or laboratory parameters. Therefore, this study was conducted to clarify these differences to support global malaria control and eradication programs. Studies reporting the occurrence of *P. ovale curtisi* and *P. ovale wallikeri* were explored in databases. Differences in proportion, clinical data, and laboratory parameters between the two species were estimated using a random-effects model and expressed as pooled odds ratios (ORs), mean difference (MD), or standardized MD depending on the types of extracted data. The difference in geographical distribution was visualized by mapping the origin of the two species. A total of 1453 *P. ovale* cases extracted from 35 studies were included in the meta-analysis. The p-value in the meta-analyses provided evidence favoring a real difference between *P. ovale curtisi* malaria cases (809/1453, 55.7%) and *P. ovale wallikeri* malaria cases (644/1453, 44.3%) (*p*: 0.01, OR 1.61, 95% CI 0.71–3.63, I^2^: 77%). Subgroup analyses established evidence favoring a real difference between *P. ovale curtisi* and *P. ovale wallikeri* malaria cases among the imported cases (*p*: 0.02, 1135 cases). The *p* value in the meta-analyses provided evidence favoring a real difference in the mean latency period between *P. ovale curtisi* (289 cases) and *P. ovale wallikeri* malaria (266 cases) (*p*: 0.03, MD: 27.59, 95% CI 1.99–53.2, I^2^: 94%), total leukocyte count (*p* < 0.0001, MD: 840, 95% CI 610–1070, I^2^: 0%, two studies) and platelet count (*p* < 0.0001, MD: 44,750, 95% CI 2900–60,500, I^2^: 32%, three studies). Four continents were found to have reports of *P. ovale* spp., among which Africa had the highest number of reports for both *P. ovale* spp. in its 37 countries, with a global proportion of 94.46%, and an almost equal distribution of both *P. ovale* spp., where *P. ovale curtisi* and *P. ovale wallikeri* reflected 53.09% and 46.90% of the continent’s proportion, respectively. This is the first systematic review and meta-analysis to demonstrate the differences in the characteristics of the two distinct *P. ovale* species. Malaria caused by *P. ovale curtisi* was found in higher proportions among imported cases and had longer latency periods, higher platelet counts, and higher total leukocyte counts than malaria caused by *P. ovale wallikeri.* Further studies with a larger sample size are required to confirm the differences or similarities between these two species to promote malaria control and effective eradication programs.

## Introduction

*Plasmodium ovale* species is a protozoan that causes benign tertian malaria, as it is a slow-growing species and rarely causes severe malaria in humans^1^. However, the most recent systematic review reported that 3% of *P. ovale* spp. malaria cases developed severe complications according to the World Health Organization (WHO) 2015 guideline, including jaundice (1.1%), severe anemia (0.88%), and pulmonary impairments (0.59%)^2^. In addition, *P. ovale* spp. infection can cause death if there is a delay in management^3,4^. Malaria caused by *P. ovale* spp. is primarily endemic in sub-Saharan Africa^5–9^, whereas it is relatively rare outside of Africa such as in some Asian countries^10–14^. Previous studies have suggested that the prevalence of *P. ovale* spp. malaria was underestimated due to its mixed infection with other *Plasmodium* species^15–18^ and misdiagnosis as *P. vivax*, which is a morphologically similar protozoan that also causes benign tertian malaria. Furthermore, a low parasitemia level in *P. ovale* spp. infection can be missed by the low sensitivity of the microscopic method^1^. Rapid diagnostic tests (RDTs) have a degree of ineffectiveness when detecting *P. ovale* spp., as their low sensitivity and specificity result in poor *P. ovale* spp. identification^19–22^. Moreover, RDTs often fail to detect *P. ovale curtisi* compared with *P. ovale wallikeri* due to the genetic variability of these two species^23^.

Polymerase chain reaction (PCR) has been recognized as the most sensitive method for detecting *P. ovale* spp. and other malaria-causing species, even in cases of a very low parasite density^24^. The PCR method has expanded the research on *P. ovale* spp. malaria and provided a far wider distribution of *P. ovale* spp. malaria cases than previously anticipated. Moreover, it allowed for the discovery of two genetically distinct sympatric species, *P. ovale curtisi* and *P. ovale wallikeri*^25^. However, the reason for the stable genetic separation between *P. ovale curtisi* and *P. ovale wallikeri* remains speculative. A previous study suggested that differences in season, region, ecology, or host red cell invasion phenotype could maintain a physical barrier between the two species^25^. Another potential reason is that these two species have accumulated mutations through a genetic drift to prevent mating or recombination^26^, and the differences in their recognition molecules that are essential for the mating process, such as the ookinete proteins, have been reported previously^27^. Nevertheless, these two species are morphologically similar and cannot be differentiated using microscopic or RDT methods, although there has been limited evidence of non-Schüffner’s stippling in *P. ovale wallikeri*-infected red blood cells^28^.

Small subunit ribosomal RNAs (*SSU rRNA*) are common amplification targets for PCR, and the PCR products can be used to confirm the species through sequencing^25,29–32^. Identification of *P. ovale* spp. depending on the characterization of *SSU rRNA* dimorphism can be compromised by mutations or genetic polymorphisms in the *SSU rRNA* gene^33^ and may result in the false identification of species. Moreover, some *P. ovale* spp. mixed infections with other *Plasmodium* spp. at a very low parasite density could be undiagnosed by the *SSU rRNA*-based PCR method^13^. Several studies have suggested that differences in genetic polymorphisms between two species are not limited to *SSU rRNA*. The genetic polymorphisms that can distinguish between *P. ovale curtisi* and *P. ovale wallikeri*, including *P. ovale* spp. tryptophan-rich antigen (*potra*)^6,7^, *P. ovale* reticulate binding protein 2 (*porbp2*)^11,25,34^, lactate dehydrogenase (*ldh*)^23^, cytochrome (*cytb* b)^12^, cytochrome oxidase subunit 1 (*cox1*)^12^, glyceraldehyde-3-phosphatase (*pog3p*)^26^, dihydrofolate reductase-thymidylate synthase (*podhfr-ts*)^12^ and the *k13* gene^35^, were initially identified to differentiate between the two *P. ovale* species.

Although several publications on *P. ovale curtisi* and *P. ovale wallikeri* malaria within and outside of endemic areas (imported cases) have been reported since 2010, there is a need for a comprehensive meta-analytic study focusing on the prevalence, proportion, distribution, and clinical and laboratory characteristics between *P. ovale curtisi* and *P. ovale wallikeri*. Therefore, this study was conducted to elucidate the differences in the characteristics of these *P. ovale* species, which would provide a better understanding of malaria caused by *P. ovale* spp. and may offer useful data for the management of patient treatment and malaria control strategies.

## Methods

The general protocol of this study followed the Preferred Reporting Items for Systematic Reviews and Meta-Analyses (PRISMA) guidelines^36^. The protocol of this systematic review is registered at PROSPERO (ID: CRD42020200985). The searches were conducted in three research databases, MEDLINE, Web of Science, and Scopus, without any restriction on language or publication date. The searches were completed on 24 July 2020. The search terms used were ‘(Plasmodium OR malaria) AND ovale AND (variant OR dimorphism OR subspecies OR curtisi OR wallikeri)’ (Table S1).

### Eligibility criteria

All types of primary studies reporting the occurrence of imported or indigenous cases of *P. ovale curtisi* and *P. ovale wallikeri* confirmed by PCR were considered as a strict eligibility criterion. If more than one study reported the occurrence of two *P. ovale* spp. in the same group of participants, the study with the higher number of *P. ovale* spp. cases was selected. Studies that were not related to species, genetic studies, review articles, case reports or case series, methodology, letters, studies without full text, and studies with data that could not be extracted were excluded from this review.

### Data selection and data extraction

Study selection and data extraction were performed independently by two authors (MK and AM). Disagreements and uncertainties of study selection were discussed and resolved by consensus. If required, the second author (FRM) was consulted for a final decision. Full texts of potentially relevant articles that matched the eligibility criteria were obtained for data extraction. Data extraction was performed by the first author (AM) and cross-checked for any inconsistency by the second author (FRM). Data from the included studies, including the name of the first author, publication year, study year, study site, age, male ratio, participants (imported or indigenous case), number of participants at enrollment, number of malaria cases, the specimen type for PCR analysis, types of PCR for identifying *P. ovale* spp., the target for PCR amplification, number of *P. ovale* spp., number of *P. ovale curtisi* and *P. ovale wallikeri* cases, parasite density, hemoglobin level, total leukocyte count, platelet count and latency period, were extracted to the pilot standardized spreadsheet for further meta-analysis.

### Quality of the included studies

The qualities of the included studies (risk of bias) were evaluated using the Newcastle–Ottawa Scale (NOS) for assessing the quality of non-randomized studies in meta-analyses with some modification for this study^37^. Each included study was judged on three broad aspects, including the selection of the study groups, the comparability of the groups, and the ascertainment of the outcome of interest. Each study was rated with stars, and the highest number of stars (five stars) indicated the highest quality. Any study rated below five stars was excluded from the present study.

### Outcomes

The primary outcome of the present study was the difference in pooled proportions between *P. ovale curtisi* and *P. ovale wallikeri*. The secondary outcome was the difference in the geographical distributions of *P. ovale curtisi* and *P. ovale wallikeri*. Tertiary outcomes were the differences in demographic profiles, latency period, and laboratory parameters between the two *P. ovale* species. The latency period was estimated from the time interval between the date of arrival and the date of illness onset in the non-endemic country.

### Data synthesis

For the primary outcome, the pooled proportion and 95% confidence interval (CI) of *P. ovale curtisi* and *P. ovale wallikeri* were estimated using the number of *P. ovale curtisi* or *P. ovale wallikeri* cases compared to the total number of *P. ovale* spp. cases. The analysis of the pooled proportion was conducted using the random-effects model on the STATA Statistical Software version 15.0 (StataCorp. 2017. Stata Statistical Software: Release 15. College Station, TX: StataCorp LLC). The differences in the proportions of *P. ovale curtisi* or *P. ovale wallikeri* were estimated using the random-effects model on Review Manager 5.3 (The Cochrane Collaboration, London, UK) available at https://training.cochrane.org/.

For the secondary outcome, the global mapping of *P. ovale* spp. malaria cases from the included studies was constructed using the online map template at https://mapchart.net/index.html. The burden score per country indicating the density of *P. ovale* spp. malaria cases in each country was calculated using the number of *P. ovale* spp. malaria cases in the included studies based on human host density. The human host density was calculated from the population per country divided by landmass. The data on population per country and landmass were sourced from https://ourworldindata.org.

For the tertiary outcomes, the pooled odds ratios (ORs), pooled mean difference (MD) or standardized mean difference (SMD), and 95% CI were estimated using the random-effects model on Review Manager (Revman) version 5.3 (Cochrane Community, UK). In the pooled MD or pooled SMD analyses, if the included studies reported median instead of mean, the mean was estimated using a protocol published elsewhere^38^. Subgroup analyses of each analysis were performed to demonstrate the differences in patients’ characteristics, PCR methods, target genes, and blood collection methods for the PCR protocol. Cochran’s Q test and I^2^ statistics were calculated to evaluate the significance and levels of heterogeneity among the included studies, respectively.

### Publication bias

The publication bias across the included studies was evaluated using funnel plots and Egger’s test, and if bias existed, a contour-enhanced funnel plot was used to demonstrate the source of the funnel plot asymmetry.

## Results

### Search results and characteristics of the included studies

**Figure 1:**
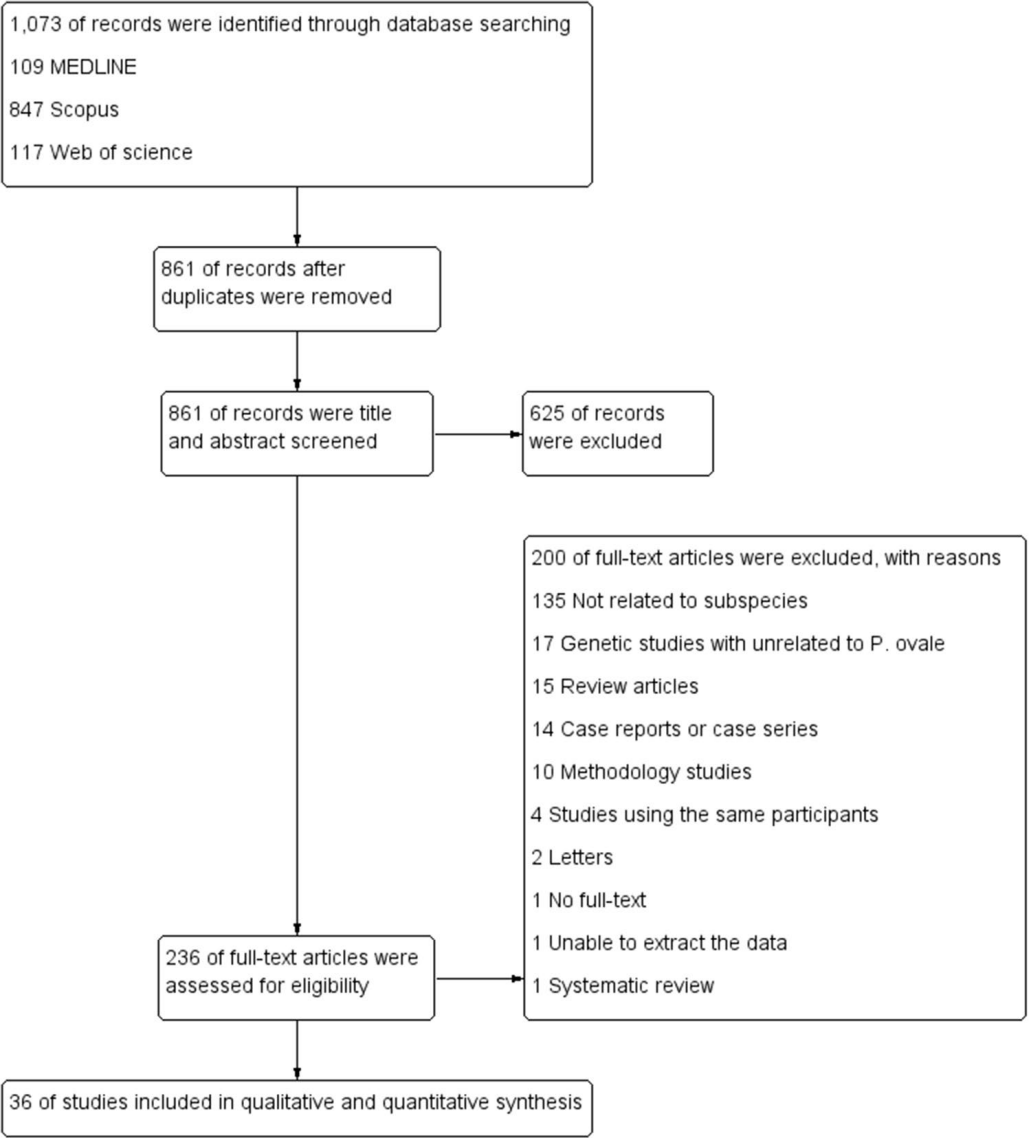
The study flow diagram.

From the searches, 1073 potentially relevant articles were identified from the MEDLINE, Web of Science, and Scopus databases. After removing the duplicate articles, 861 articles qualified for the title and abstract review phase. After reviewing the title and abstract, 625 articles were excluded, and 236 articles were selected for full-text screening. After screening the full texts, 200 articles were excluded due to the following reasons: 135 not related to species, 17 genetic studies unrelated to *P. ovale* spp., 15 review articles, 14 case reports or case series, 10 methodology studies, 4 studies using the same participants, 2 letters, 1 with full-text that could not be retrieved, 1 with data that could not be extracted and 1 systematic review. Finally, a total of 36 studies^5–15,23,25,26,28–32,34,35,39–53^ were found to meet the eligibility criteria and thus included in the present analysis (Fig. 1).

All included studies were published between 2010, which was the year of the first identified distinct *P. ovale* spp. by Sutherland et al.^25^, and 2020. The majority of included studies (14/36, 38.9%) were conducted in African countries (1 in Angola^9^, 1 in Equatorial Guinea^26^, 2 in Ethiopia^5,8^, 2 in Gabon^43,51^, 1 in Ghana^15^, 1 in Kenya^46^, 1 in Namibia^44^, 2 in the Republic of Congo/Congo^26,42^, 2 in Senegal^6,7^ and 1 in Uganda^26^), Asian countries (9 in China^12,14,32,35,39,49,50,52,53^, 2 in India^10,11^, 1 in Bangladesh^41^, 1 in Myanmar^12^ and 1 in Thailand^13^), Europe (3 in Italy^29–31^, 2 in France^23,45^, 2 in Spain^47,48^, 2 in the United Kingdom^25,34^ and 1 in Germany^40^) and Northern America (1 in Canada^28^). Most of the included studies^23,25,28,30–32,34,35,39,40,45,47–50,52,53^ (19/36, 52.8%) reported indigenous cases of malaria caused by *P. ovale curtisi* or *P. ovale wallikeri*, and the remaining studies (17/36, 47.2%) reported imported cases.

A study conducted by Chu et al.^39^ was not included for meta-analysis but was included for assessing the geographical distribution of *P. ovale* spp. cases, as that study sample overlapped with that of the study conducted by Cao et al.^32^; however, the study conducted by Chu et al.^39^ reported more cases with different origins of imported *P. ovale* spp. than the study conducted by Cao et al.^32^. The study conducted by Cao et al.^32^ was used for the meta-analysis because it reported more clinical characteristics and laboratory data, but it was not included in the geographical distribution map. All the characteristics of the included studies are listed in Table S2. The qualities of the included studies were evaluated using NOS and presented in Table S3.

### Geographical distribution and burden score per country for malaria caused by *P. ovale curtisi* and *P. ovale wallikeri*

**Figure 2:**
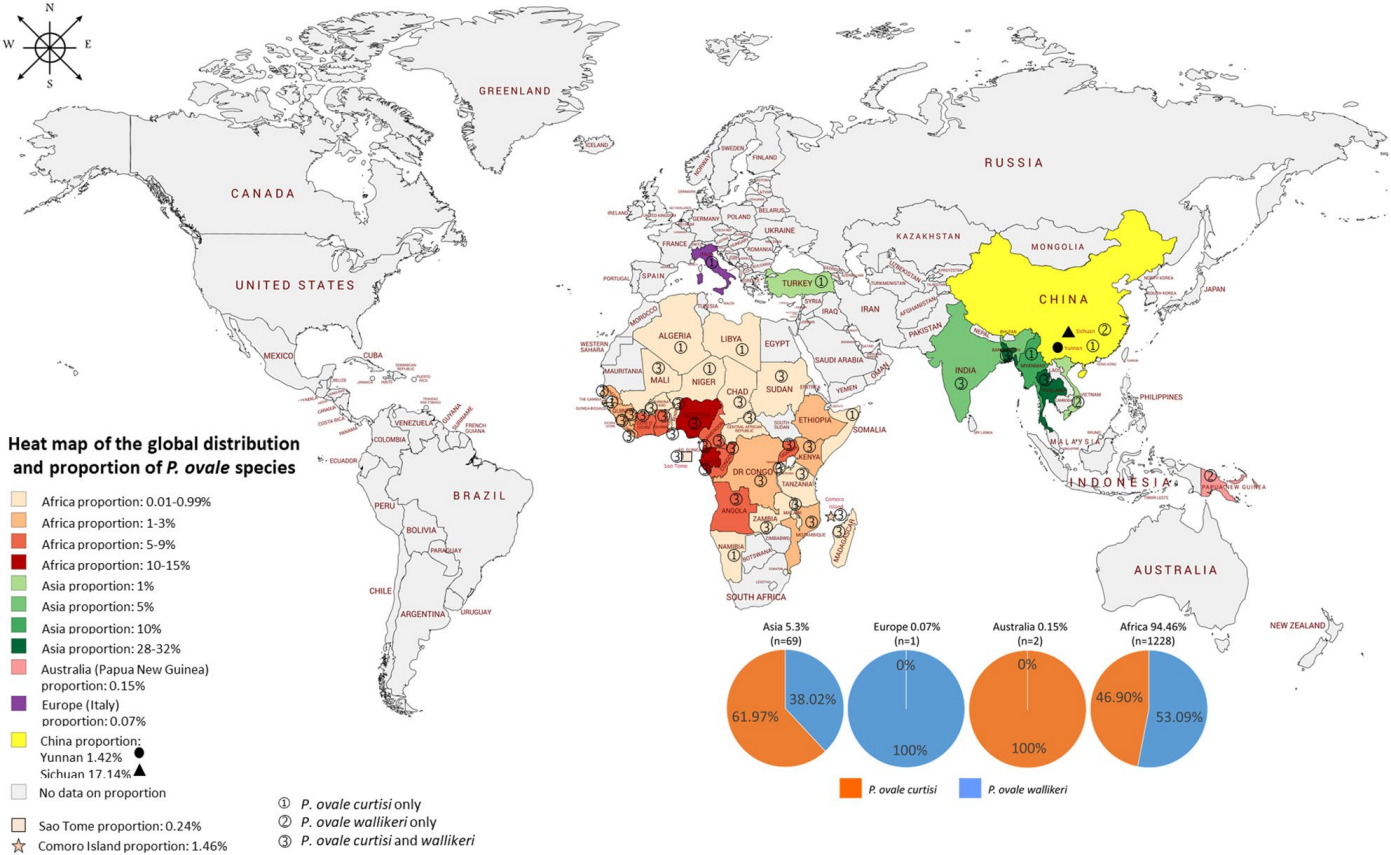
The geographical distribution of *P. ovale* spp. The map was generated by authors using the map freely available at https://mapchart.net/. Authors are allowed to use, edit and modify any map created with mapchart.net for publication freely by adding the reference to mapchart.net. The project of is licensed under a Creative Commons Attribution-ShareAlike 4.0 International License.

A total of 1300 *P. ovale* spp. identified from 35 studies were used to construct the geographical distribution map of malaria caused by *P. ovale curtisi* and *P. ovale wallikeri* (Fig. 2). Based on data from the included studies, four continents were found to have reports of *P. ovale* species. From the pooled analysis of *P. ovale* spp., 94.46% of cases were reported from 37 countries of Africa, 5.30% of cases were reported from eight territories of Asia (*P. ovale wallikeri*, 60.86%) and 0.07% of *P. ovale curtisi* and 0.15% of *P. ovale wallikeri* malaria cases were reported from Europe and Australia, respectively. Globally, there was an almost equal distribution of both *P. ovale* spp., where *P. ovale curtisi* and *P. ovale wallikeri* reflected 53.09% and 46.90%, respectively.

In the African region, most of the *P. ovale curtisi* malaria cases were reported from Nigeria (107/680, 15.7%), Equatorial Guinea (82/680, 12.1%), and Gabon (82/680, 12.1%), whereas most of the *P. ovale wallikeri* malaria cases were reported from Equatorial Guinea (75/620, 12.1%), Nigeria (74/620, 11.9%) and Cameroon (55/620, 8.9%). In Asian countries, the majority of *P. ovale curtisi* malaria cases were reported from Bangladesh (10/680, 1.47%), Myanmar (7/680, 1.03%), and Thailand (5/680, 0.74%), whereas the majority of *P. ovale wallikeri* malaria cases were reported from Thailand (15/620, 2.42%), Bangladesh (13/620, 2.1%) and China (Sichuan Province) (12/620, 1.94%). One case of indigenous *P. ovale curtisi* malaria in a non-endemic country was reported in Italy^29^. Mixed infections of *P. ovale curtisi* and *P. ovale wallikeri* were also identified, including 9 cases in a study conducted by Woldearegai et al.^51^, 4 cases in a study conducted by Groger et al.^43^ and 1 case in a study conducted by Fuehrer et al.^41^.

The burden score per country established the following top five countries with the host (human) burden score for *P. ovale* spp.: Gabon with a staggering burden score of 15.750, the Republic of Congo/Congo (4.250), Angola (3.440), Equatorial Guinea (3.271), and Cameroon (1.436) (Table S4).

### Difference in the proportion of *P. ovale curtisi* and *P. ovale wallikeri* malaria cases

**Figure 3:**
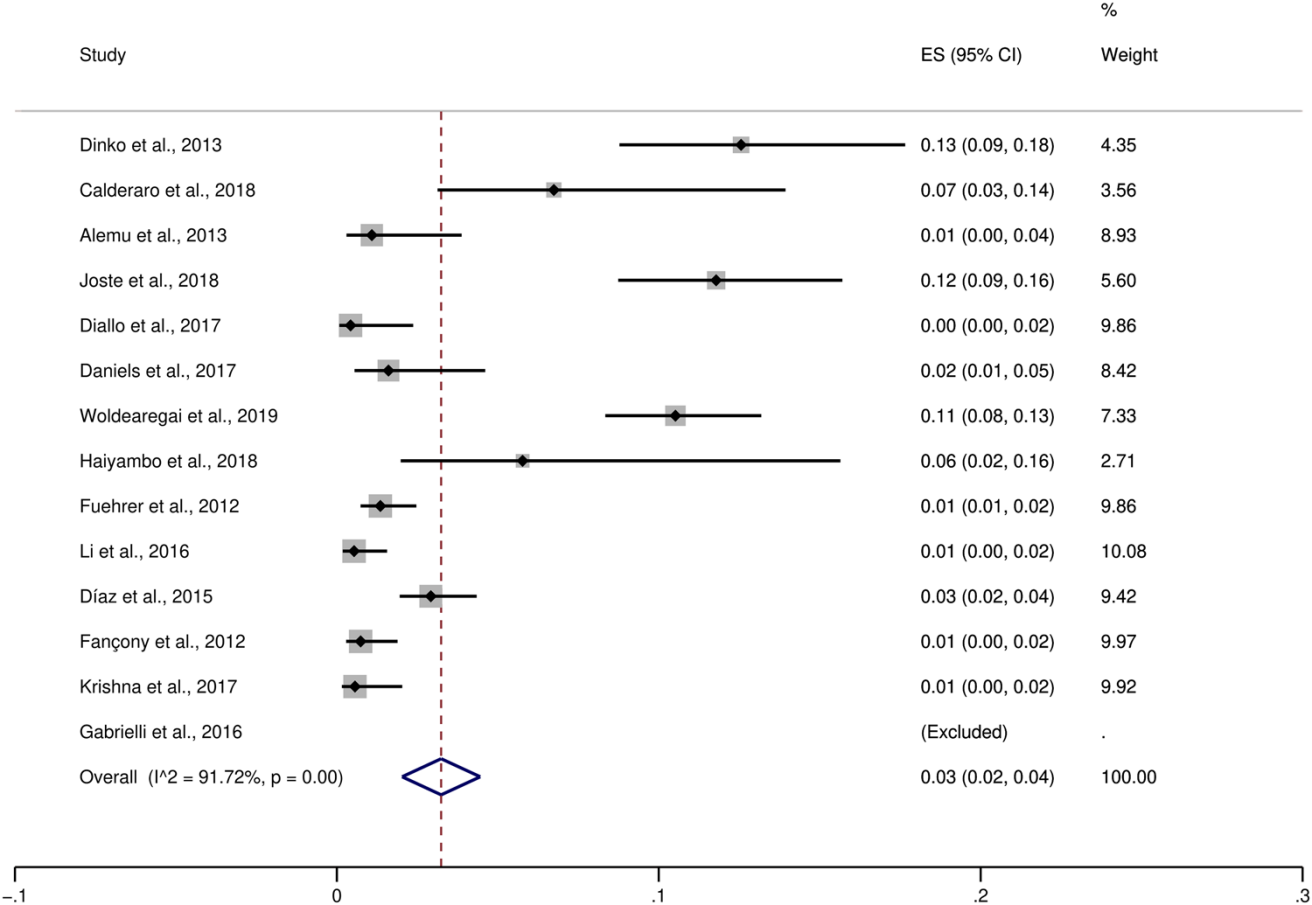
The pooled proportion of *P. ovale curtisi* malaria. *ES* estimated proportion, *CI* confidence interval, *Random* random-effects model. The study conducted by Gabrielli et al. (2016) was excluded from the analysis because no *P. ovale curtisi* case was reported.

**Figure 4:**
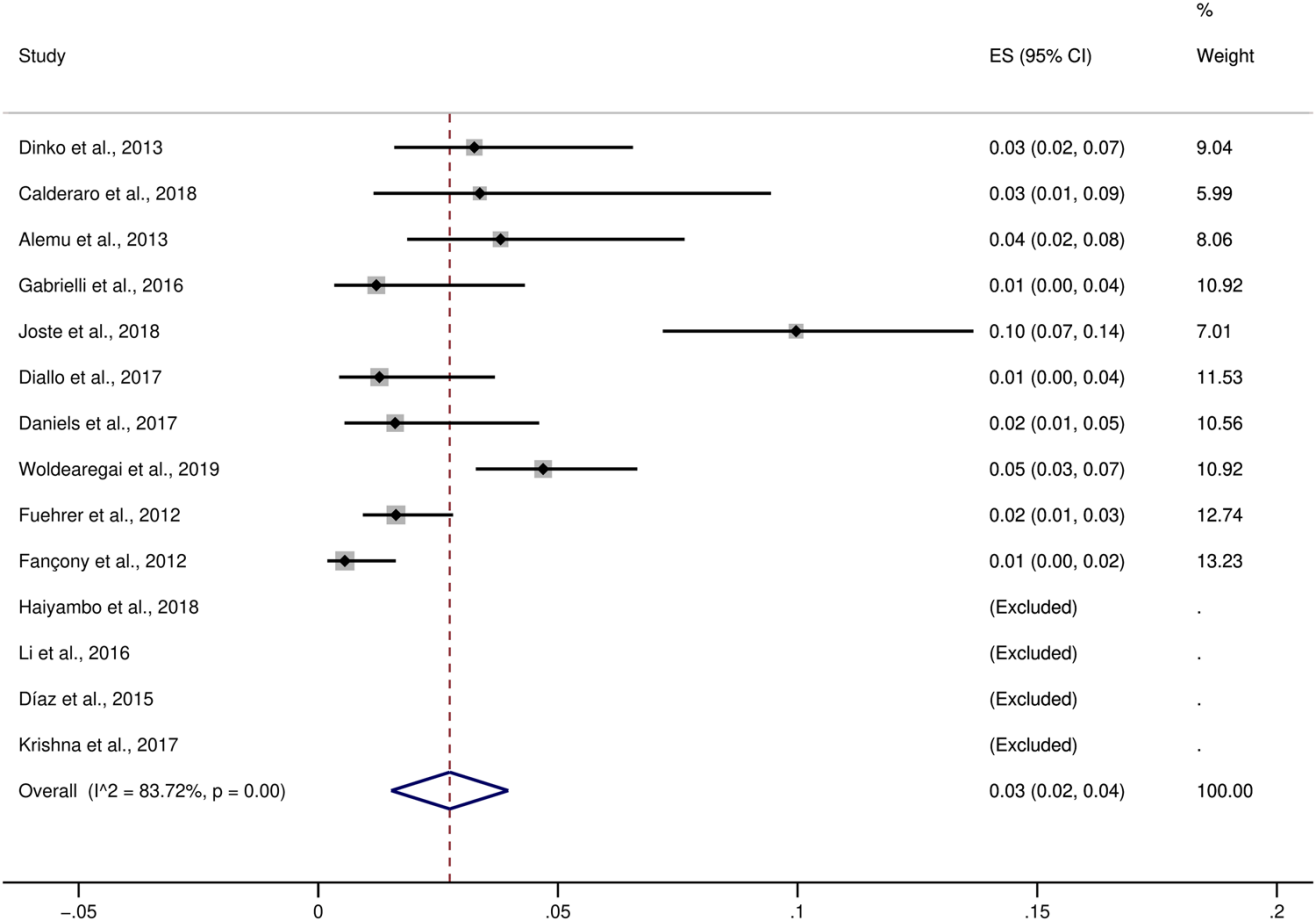
The pooled proportion of *P. ovale wallikeri* malaria. *ES* estimated proportion, *CI* confidence interval, *Random* random-effects model. The studies conducted by Haiyambo et al. (2018), Li et al. (2016), Díaz et al. (2015), and Krishna et al. (2017) were excluded from the analysis because no *P. ovale curtisi* case was reported.

**Figure 5:**
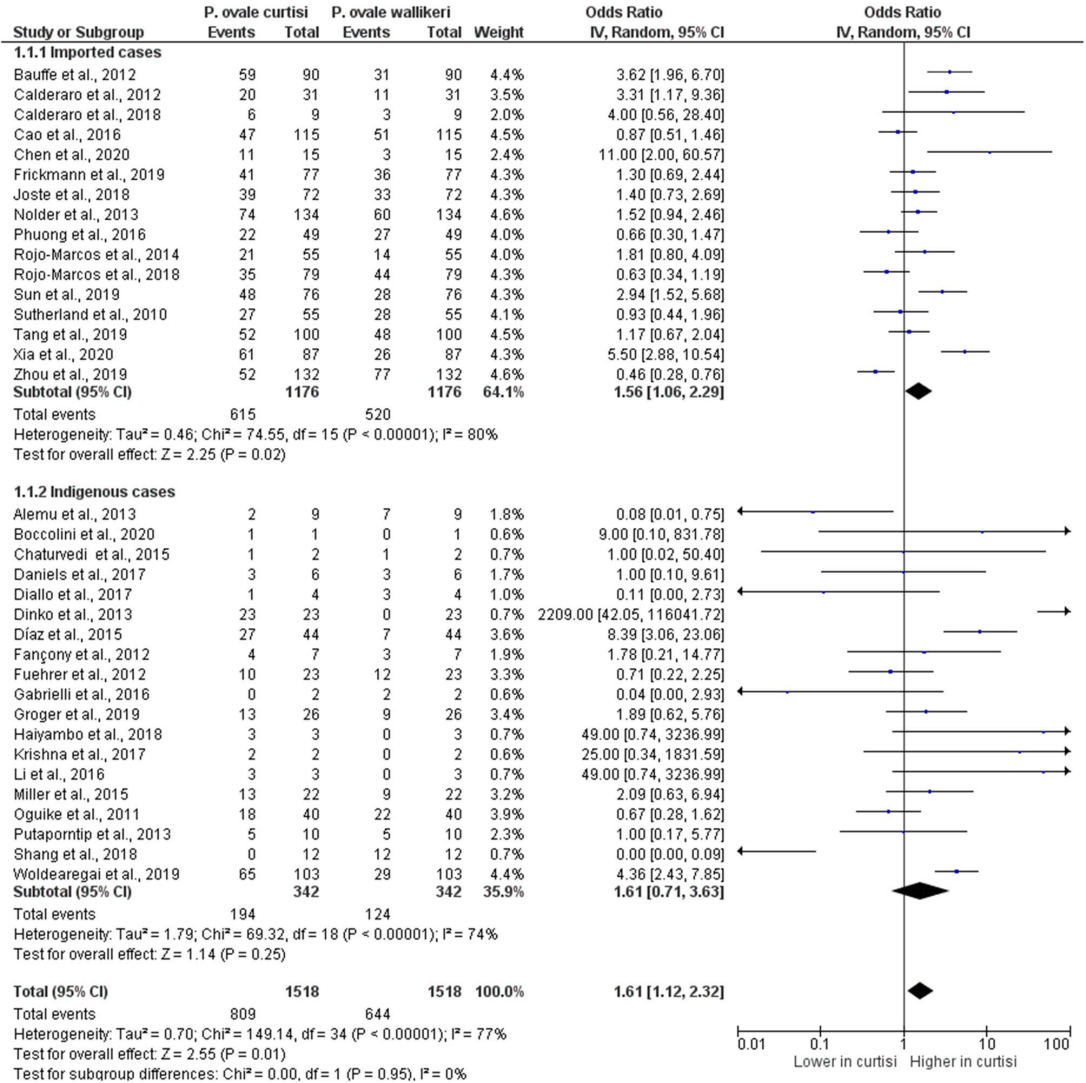
The difference in proportion between *P. ovale curtisi* and *P. ovale wallikeri* malaria among imported and indigenous cases. *IV* inverse variance, *CI* confidence interval, *Event* Number of *P. ovale curtisi* or *P. ovale wallikeri* cases, *Random* random-effects model, *Total* number of all *P. ovale* spp. cases, Lower in *curtisi*: The proportion of *P. ovale curtisi* cases was lower than that of *P. ovale wallikeri* cases. Higher in *curtisi*: The proportion of *P. ovale curtisi* cases was higher than that of *P. ovale wallikeri* cases.

The pooled proportion of *P. ovale curtisi* malaria cases compared to all *P. ovale* spp. malaria cases were estimated, which revealed a pooled proportion of *P. ovale curtisi* malaria cases of 3% with a large heterogeneity between studies (95% CI 2–4%, I^2^: 91.7%, 13 studies) (Fig. 3). The highest proportions of *P. ovale curtisi* malaria cases were reported in studies conducted by Dinko et al.^15^ (13%, 95% CI 9–18%), Joste et al.^45^ (12%, 95% CI 9–16%) and Woldearegai et al.^51^ (11%, 95% CI 8–13%). Regarding the pooled proportion of *P. ovale wallikeri* malaria cases compared to all *P. ovale* spp. cases, there was a proportion of 3% of *P. ovale wallikeri* malaria cases with a large heterogeneity between studies (95% CI 2–4%, I^2^: 83.7%, 10 studies) (Fig. 4). The highest proportion of *P. ovale wallikeri* malaria cases was reported in a study conducted by Joste et al. (2018) (10%, 95% CI 7–14%). The difference in the pooled proportion between *P. ovale curtisi* and *P. ovale wallikeri* malaria cases was analyzed using all 35 studies. Overall, the p-value in the meta-analyses provided evidence favoring a real difference between the proportion of *P. ovale curtisi* malaria cases (809/1453, 55.7%) and that of *P. ovale wallikeri* malaria cases (644/1453, 44.3%) (*p*: 0.01, OR: 1.61, 95% CI 1.12–2.32, I^2^: 77%, 35 studies) (Fig. 5).

Subgroup analyses were performed to explore the differences in the subgroup of *P. ovale* spp. malaria cases, which helped examine the source of heterogeneity across the included studies. The subgroup analysis of *P. ovale* spp. malaria cases provided evidence favoring a real difference between the proportion of *P. ovale curtisi* malaria cases (615 cases) and that of *P. ovale wallikeri* malaria cases (520 cases) among imported cases (*p*: 0.02, OR: 1.56, 95% CI 1.06–2.29, I^2^: 80%), whereas no difference was observed in the proportion between *P. ovale curtisi* malaria cases (194 cases) and *P. ovale wallikeri* malaria cases (124 cases) among the indigenous cases (*p*: 0.25, OR: 1.61, 95% CI 0.71–3.63, I^2^: 74%) (Fig. 5).

### Difference in demographic data between *P. ovale curtisi* and *P. ovale wallikeri* malaria cases

**Figure 6:**
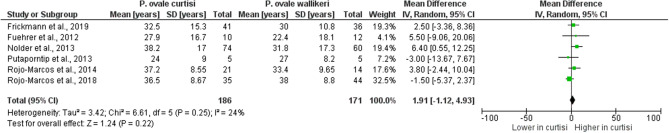
The difference in age between *P. ovale curtisi* and *P. ovale wallikeri* malaria. *IV* inverse variance, *CI* confidence interval, *SD* standard deviation, *Random* random-effects model, *Total* number of all *P. ovale* spp. cases, Lower in *curtisi*: The mean age of *P. ovale curtisi* cases was lower than that of *P. ovale wallikeri* cases. Higher in curtisi: The mean age of *P. ovale curtisi* cases was higher than that of *P. ovale wallikeri* cases.

**Figure 7:**
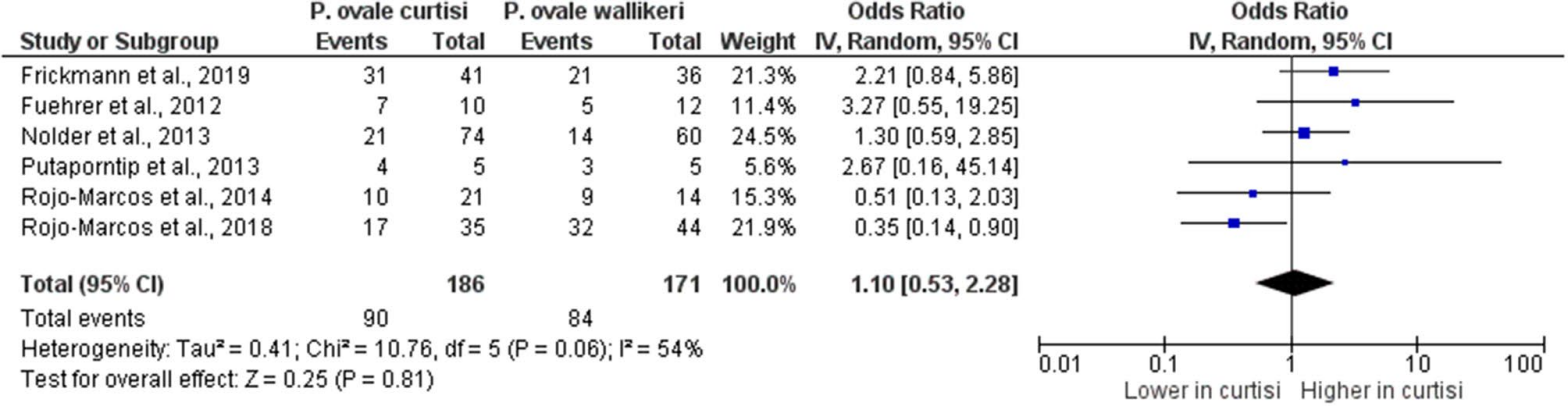
The difference in gender between *P. ovale curtisi* and *P. ovale wallikeri* malaria. *IV* inverse variance, *CI* confidence interval, *Event* number of male patients with *P. ovale curtisi* or *P. ovale wallikeri*, *Random* random-effects model, *Total* number of all *P. ovale* spp. cases, Lower in *curtisi*: The proportion of male patients with *P. ovale curtisi* cases was lower than that of *P. ovale wallikeri* cases. Higher in *curtisi*: The proportion of male patients with *P. ovale curtisi* cases was higher than that of *P. ovale wallikeri* cases.

The differences in demographic data, including age and gender, between *P. ovale curtisi* and *P. ovale wallikeri* malaria cases were analyzed. Six studies^13,34,40,41,47,48^ that reported the mean or median age of patients with *P. ovale* spp. malaria were included in the analysis. The results of this analysis revealed no difference in the mean age between patients with *P. ovale curtisi* malaria (186 cases) and those with *P. ovale wallikeri* malaria (171 cases) (*p*: 0.22, MD: 1.91, 95% CI 1.12–4.93, I^2^: 24%) (Fig. 6). Six studies^13,34,40,41,47,48^ that reported the mean or median age of patients with *P. ovale* spp. malaria was included in the analysis. The results of the analysis of gender, between patients with *P. ovale curtisi* malaria (186 cases) and those with *P. ovale wallikeri* malaria (171 cases), revealed no difference (*p*: 0.81, OR: 1.10, 95% CI 0.53–2.28, I^2^: 54%) (Fig. 7).

### Difference in latency period and laboratory parameters between *P. ovale curtisi* and *P. ovale wallikeri* malaria cases

**Figure 8:**
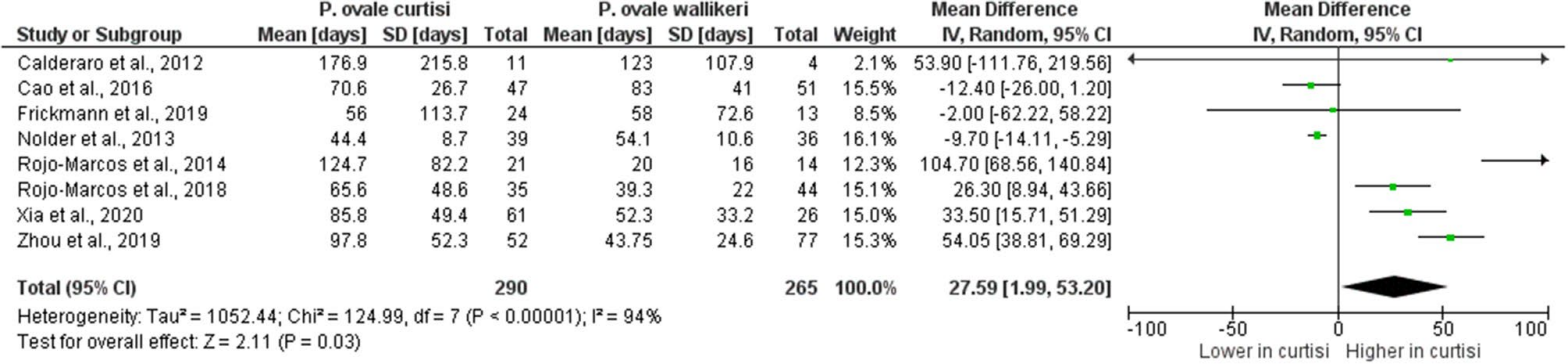
The difference in latency period between *P. ovale curtisi* and *P. ovale wallikeri* malaria. *IV* inverse variance, *CI* confidence interval, *SD* standard deviation, *Random* random-effects model, *Total* number of all *P. ovale* spp. cases, Shorter in *curtisi*: The mean latency period of *P. ovale curtisi* cases was shorter than that of *P. ovale wallikeri* cases. Longer in *curtisi*: The mean latency period of *P. ovale curtisi* cases was longer than that of *P. ovale wallikeri* cases.

The latency period in each study was calculated from the period in days between the last date in the malaria-endemic country and presentation to the hospital in the non-endemic country. Eight studies^30,32,34,40,47,48,52,53^ reported the mean latency period (days) of *P. ovale* spp. and were included in the analysis. The mean latency period of *P. ovale curtisi* ranged from 44.4 to 176.9 days, whereas the mean latency period of *P. ovale wallikeri* ranged from 20 to 123 days. The difference in the mean latency period (days) between *P. ovale curtisi* and *P. ovale wallikeri* malaria was analyzed, which showed a longer mean latency period of *P. ovale curtisi* (290 cases) than that of *P. ovale wallikeri* (265 cases) reported in four studies^47,48,52,53^, and a shorter mean latency period of *P. ovale curtisi* than that of *P. ovale wallikeri* was demonstrated in a study reported by Nolder et al.^34^. Overall, the meta-analysis provided evidence favoring a real difference in the mean latency period between *P. ovale curtisi* (289 cases) and *P. ovale wallikeri* (266 cases) malaria cases (*p*: 0.03, MD: 27.59, 95% CI 1.99–53.2, I^2^: 94%) (Fig. 8).

**Figure 9:**
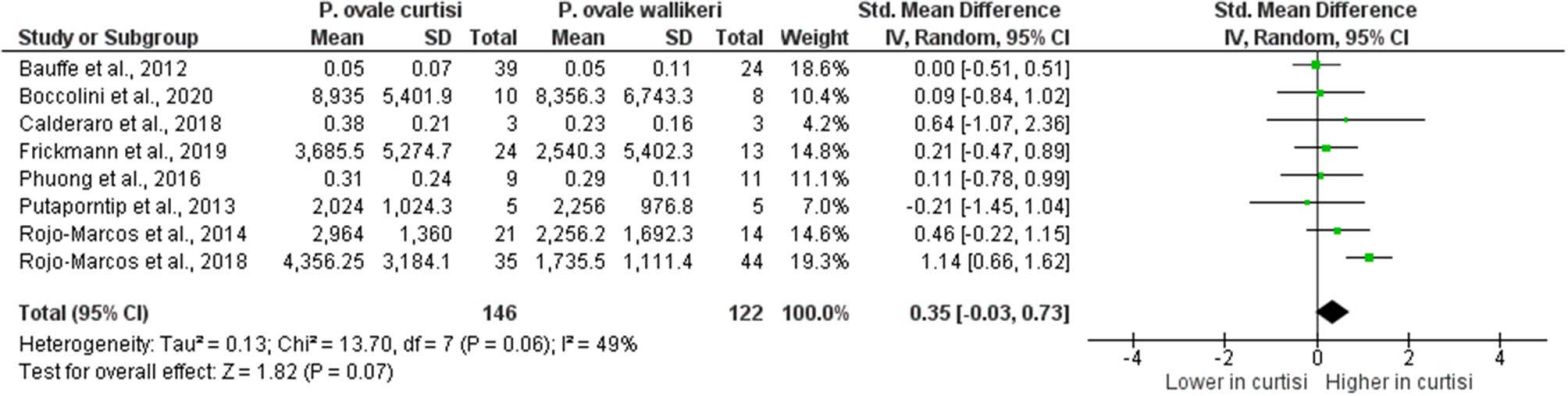
The difference in parasite density between *P. ovale curtisi* and *P. ovale wallikeri* malaria. *IV* inverse variance, *CI* confidence interval, *SD* standard deviation, *Std. mean difference* Standard mean difference (SMD), *Random* random-effects model, *Total* number of all *P. ovale* spp. cases, Lower in *curtisi*: The SMD of parasite density in *P. ovale curtisi* cases was lower than that in *P. ovale wallikeri* cases. Higher in *curtisi*: The SMD of parasite density in *P. ovale curtisi* cases was higher than that in *P. ovale wallikeri* cases. Mean of parasite density is in parasites per microliter (parasites/µL).

**Figure 10:**
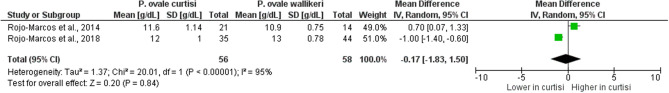
The difference in hemoglobin level between *P. ovale curtisi* and *P. ovale wallikeri* malaria. *IV* inverse variance, *CI* confidence interval, *SD* standard deviation, *Random* random-effects model, *Total* number of all *P. ovale* spp. cases, Lower in *curtisi*: The mean hemoglobin of *P. ovale curtisi* cases was lower than that of *P. ovale wallikeri* cases. Higher in *curtisi:* The mean hemoglobin level of *P. ovale curtisi* cases was higher than that of *P. ovale wallikeri* cases.

**Figure 11:**
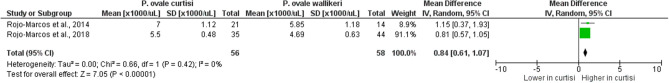
The difference in leukocyte count between *P. ovale curtisi* and *P. ovale wallikeri* malaria. *IV* inverse variance, *CI* confidence interval, *SD* standard deviation, *Random* random-effects model, *Total* number of all *P. ovale* spp. cases, Lower in *curtisi*: The mean leukocyte counts of *P. ovale curtisi* cases was lower than that of *P. ovale wallikeri* cases. Higher in *curtisi*: The mean leukocyte counts of *P. ovale curtisi* cases were higher than those of *P. ovale wallikeri* cases.

**Figure 12:**
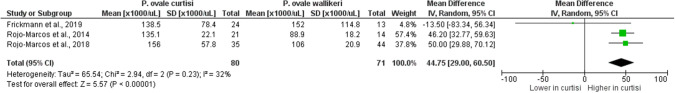
The difference in platelet counts between *P. ovale curtisi* and *P. ovale wallikeri* malaria. *IV* inverse variance, *CI* confidence interval, *SD* standard deviation, *Random* random-effects model, *Total* number of all *P. ovale* spp. cases, Lower in *curtisi*: The mean platelet counts of *P. ovale curtisi* cases were lower than those of *P. ovale wallikeri* cases. Higher in *curtisi*: The mean platelet counts of *P. ovale curtisi* cases were higher than those of *P. ovale wallikeri* cases.

For analyzing the differences in parasite density, hemoglobin, and platelet counts between *P. ovale curtisi* and *P. ovale wallikeri* malaria cases, eight studies^13,23,28,29,31,40,47,48^ that reported the mean or median parasite density of *P. ovale* spp. were included in the analysis. The results revealed no difference in the mean parasite density between *P. ovale curtisi* (146 cases) and *P. ovale wallikeri* (122 cases) malaria cases (*p*: 0.07, SMD: − 0.03, 95% CI − 0.03–0.73: I^2^: 49%) (Fig. 9). The meta-analysis provided evidence favoring a real difference in the mean parasite density between *P. ovale curtisi* and that of *P. ovale wallikeri* in the study conducted by Rojo–Marcos et al. (SMD: 1.14, 95% CI − 0.66–1.62)^47^. Two studies^47,48^ that reported the mean hemoglobin level of *P. ovale* spp. were included in the analysis, and no difference was found in the mean hemoglobin (g/dL) level between *P. ovale curtisi* (56 cases) and *P. ovale wallikeri* (58 cases) malaria cases (p: 0.84, MD: − 0.17, 95% CI − 1.83–1.50: I^2^: 95%) (Fig. 10). In cases of *P. ovale curtisi* malaria, there was a lower mean hemoglobin level than that in cases of *P. ovale wallikeri* malaria as demonstrated in the study reported by Rojo–Marcos et al. (MD: − 1.00, 95% CI − 1.83–1.50)^47^. Two studies^47,48^ that reported the mean total leukocyte count of *P. ovale* spp. were included in the analysis. The meta-analysis provided evidence favoring a real difference in the mean total leukocyte count between *P. ovale curtisi* (56 cases) and *P. ovale wallikeri* (58 cases) malaria cases (*p* < 0.0001, MD: 840, 95% CI 610–1,070: I^2^: 0%) (Fig. 11). This difference in the mean total leukocyte count was reported in studies conducted by Rojo–Marcos et al.^47,48^. Three studies^40,47,48^ that reported the mean platelet count of *P. ovale* spp. were included in the analysis, and the meta-analysis provided evidence favoring a real difference in the mean platelet count between *P. ovale curtisi* (80 cases) and *P. ovale wallikeri* (70 cases) malaria cases (*p* < 0.0001, MD: 44,750, 95% CI 29,000–60,500: I^2^: 0%) (Fig. 12). In cases of *P. ovale wallikeri* malaria, a higher mean platelet count was detected than that in cases of *P. ovale curtisi* malaria in the studies reported by Rojo–Marcos et al.^47,48^.

### Differences in PCR methods, target genes, and blood samples for identifying *P. ovale* spp. malaria cases

Subgroup analyses were performed to examine the differences in the subgroup of *P. ovale* spp. malaria cases, which helped explore the source of heterogeneity across the included studies. A total of 35 studies were included in the subgroup analyses. The subgroup analysis of PCR methods revealed a higher proportion of *P. ovale curtisi* malaria cases (251 cases) than that of *P. ovale wallikeri* malaria cases (170 cases) when using a real-time PCR method (*p*: 0.006, OR: 2.24, 95% CI 1.26–3.99, I^2^: 74%). There was no difference in the proportion of *P. ovale curtisi* (373 cases) and *P. ovale wallikeri* (300 cases) malaria cases when using the nested PCR method (*p*: 0.09, OR: 1.75, 95% CI 0.92–3.34, I^2^: 82%). There was also no significant difference in the proportion of *P. ovale curtisi* and *P. ovale wallikeri* malaria cases in other subgroups (*p* > 0.05) (Supplementary Figure 1).

The subgroup analysis of target genes for PCR methods demonstrated a higher proportion of *P. ovale curtisi* (257 cases) malaria cases than that of *P. ovale wallikeri* malaria cases (181 cases) observed in PCR amplification of the *SSU rRNA* and other target genes (*p*: 0.02, OR: 1.91, 95% CI 1.10–3.32, I^2^: 75%). There was no difference in the proportion of *P. ovale curtisi* (388 cases) and *P. ovale wallikeri* (334 cases) malaria cases when only the *SSU rRNA* target gene was PCR-amplified (*p*: 0.16, OR: 1.50, 95% CI 0.86–2.62, I^2^: 82%). There was also no difference in the proportion of *P. ovale curtisi* (164 cases) and *P. ovale wallikeri* (117 cases) malaria cases when non-*SSU rRNA* target genes were PCR-amplified (*p*: 0.16, OR: 1.50, 95% CI 0.86–2.62, I^2^: 82%) (Supplementary Figure 2).

The subgroup analysis of blood samples disclosed a higher proportion of *P. ovale curtisi* malaria cases (121 cases) than that of *P. ovale wallikeri* malaria cases (51 cases) in dried blood spots used for DNA extraction (*p*: 0.03, OR: 3.86, 95% CI 1.12–13.31, I^2^: 73%). There was no difference in the proportion of *P. ovale curtisi* (250 cases) and *P. ovale wallikeri* (204 cases) malaria cases in venous blood samples used for DNA extraction (*p*: 0.17, OR: 1.49, 95% CI 0.85–2.60, I^2^: 75%) (Supplementary Figure 3).

### Publication bias

**Figure 13:**
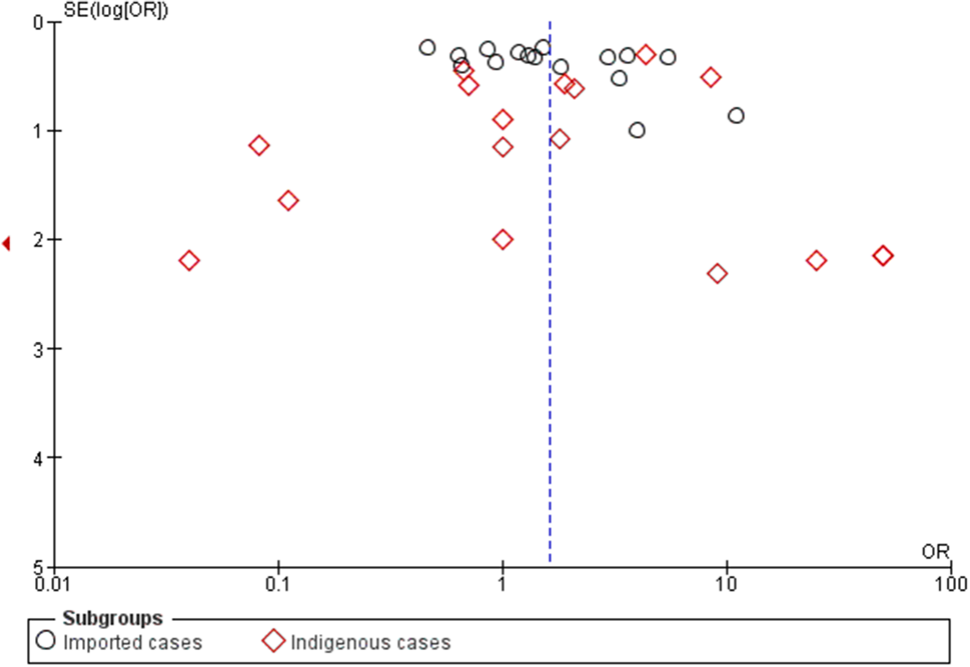
Funnel plot. *SE* standard error, *OR* odds ratio.

The publication bias across the included studies was evaluated using the funnel plot and Egger’s test. The results of Egger’s test demonstrated that no small-study effects were found (*p*: 0.09, coefficient: 2.21, standard error: 1.27, *t*: 1.74), indicating the absence of potential publication bias across the included studies (Fig. 13).

## Discussion

*P. ovale* spp. infection is increasingly observed among travelers who return from *P. ovale*-endemic areas. However, due to its lower mortality (0.15%)^2^ than that of malaria caused by other non-*P. falciparum* spp. such as *P. vivax* and *P. malariae*, and also due to mixed infection^54–57^, there has been limited focus on *P. ovale* spp. in malaria research. The first identification of the species *P. ovale wallikeri* and *P. ovale curtisi* was reported by Sutherland et al.^25^. Since then, differences in morphology, clinical characteristics, laboratory parameters and genetic differences between the two species have been observed in Africa^5–9,15,26,42–44,46,51^, Asia^10–14,32,35,39,41,49,50,52,53^, Europe^23,25,29–31,34,40,45,47,48^, and North America^28^. This widespread distribution of *P. ovale* spp. has necessitated the expansion of its research to other parts of the world, due to the increasing numbers of imported cases in non-endemic countries^23,25,28,30–32,34,35,39,40,45,47–50,52,53^.

Overall, the pooled prevalence demonstrated a similar proportion of *P. ovale curtisi* and *P. ovale wallikeri* malaria cases (both 3%), indicating a similar geographical distribution among these two species. However, the meta-analysis of the proportion between these two species demonstrated a significantly higher proportion of *P. ovale curtisi* than that of *P. ovale wallikeri* malaria cases. The subgroup analysis of *P. ovale* spp. malaria cases showed that imported cases were related to a higher proportion of *P. ovale curtisi* malaria cases. It was observed that a higher proportion of *P. ovale curtisi* malaria cases was predominantly reported from travelers returning to France^23^, Italy^30^, and China^35,49,52^. Although the majority of *P. ovale* spp. malaria cases were imported from endemic countries in Africa, some studies have reported that *P. ovale curtisi* malaria originated in some Asian countries, including Myanmar^12,35^, India^10,11^, and Turkey^52^, whereas *P. ovale wallikeri* malaria originated and predominated in Papua New Guinea^25,40^, Bangladesh^41,52^, China^14^, Thailand^13,25^ and Vietnam^25^; a sympatric distribution of *P. ovale curtisi* and *P. ovale wallikeri* was maintained in Western Africa and Asia^25,30,33,58^.

It is interesting to ponder as to why some countries reported only a single species of *P. ovale* spp. despite being surrounded by countries with both *P. ovale* spp., as is the case of Myanmar in Asia having only *P. ovale curtisi*. A similar situation was observed in Guinea-Bissau, Algeria, Niger, Libya, Somalia, and Namibia in Africa as having only *P. ovale curtisi*. This is a curious observation as all these countries are connected by land unlike the case of Sao Tome and the Comoro Islands, which despite being located off the coast of Africa reported the presence of both *P. ovale curtisi* and *P. ovale wallikeri*. However, it can be observed that for both cases of Sao Tome and the Comoro Islands, the countries in the closest proximity reported the presence of both *P. ovale* species. It is also interesting to note that there was an absence of reports of *P. ovale* spp. in Laos, Cambodia, and Nepal in Asia and Gambia, South Sudan, and Rwanda in Africa, all of which are surrounded by countries reporting the cases of both *P. ovale* species. The absence of *P. ovale* spp. has not been investigated and confirmed in these countries and no publications are available, indicating that perhaps relevant research has not been conducted in these areas. The incidence of parasitic infection significantly depends on different factors that support its successful transmission to a healthy host to complete its life cycle. In the case of *P. ovale* spp., the mosquito vector that can pass the protozoan to another host through blood meals is important when considering the spread of malaria cases in a given population. The host density is important in the algorithm of transmission and the eventual survival of malarial parasite that relies heavily on the contact between infected hosts, vectors and susceptible hosts^59^. This translates that densely populated countries as potentially being more susceptible to developing more cases than less densely populated territories. Three Asian countries that have an unbalanced landmass-to-population ratio due to overpopulation, viz., Sichuan Province of China (1:433), Thailand (1:136), and Bangladesh (1:1252), registered the maximum number of *P. ovale* spp. malaria cases detected in Asia. A similar situation was observed in the African countries of the Comoro Islands (1:457), Sierra Leone (1:108), Ethiopia (1:112), Uganda (1:210), Ivory Coast (1:80), Ghana (1:133), and Nigeria (1:220), which also registered a greater number of malaria cases caused by *P. ovale* species. Although this concept of host density may support the *P. ovale* species malaria cases in the mentioned countries, it may not be true for other countries that share the same overpopulation statistics and registered only ten cases or fewer. These countries include Vietnam (1:311), India (1:461), Burundi (1:448), Sao Tome (1:223), Togo (1:148) and Malawi (1:197). This further explains that overpopulation alone may not be a primary factor in the incidence of malarial infection; in this case, *P. ovale* spp., but rather, involves several contributing factors. Several factors such as climate change, elevation, and vector control programs are important areas to be considered. The results of the present study are consistent with those of previous studies conducted in Africa on the incidence of malaria in region^60^. The host (human) burden score per country, when arranged in ascending order, showed that the top five countries with the highest scores for *P. ovale* spp. were Gabon with a staggering burden score of 15.750, followed by the Republic of Congo/Congo (4.250), Angola (3.440), Equatorial Guinea (3.270) and Cameroon (1.436). Interestingly, mixed infections of *P. ovale curtisi* and *P. ovale wallikeri* were identified only in Bangladesh and Gabon. Given these observations, Gabon and Bangladesh are countries with established research centers and active antimalarial research, such as the Centre de Recherches Médicale de Lambaréné (CERMEL)^61^ and the Centre International de Recherches Medicales de Franceville (CIRMF) in Gabon^62^, the international health research organization (International Centre for Diarrhoeal Disease Research, Bangladesh or icddr,b [sic]) in Bangladesh^63^. The presence of a regional research center may partially explain why Bangladesh had one of the lowest host (human) burden scores (0.018) despite having the highest host (human) density (1252).

These data provide interesting insights into the number of reported cases of *P. ovale* malaria per country when compared to the host (human) density (1 km^2^: number of individuals) that provides the computed host (human) burden score per country (Table S4). As demonstrated by the computed host (human) burden score, the top ten countries with the highest scores were all located in the African continent, with Gabon, the Republic of Congo/Congo, Angola, Equatorial Guinea, and Cameroon ranking 1st to 5th, respectively. This high burden score may also be due to the inherently high overall malaria incidence on the African continent. Interestingly, these countries had a greater number of reported *P. ovale* spp. malaria cases than the host (human) density computed for each country, suggesting either a high burden of *P. ovale* spp. malaria or possibly a bias of some sort for high reports of the same. Whatever the case may be, a deeper investigation of the occurrence of these high numbers of cases may provide interesting and useful perspectives for malaria case-finding and control. The same can be stated for countries with the lowest number of *P. ovale* spp. malaria cases, which were predominantly found in the Asian and European continents. However, these data should be interpreted with caution as the population per country (relative to landmass) was considered as a whole irrespective of the urban or rural population distribution and that humans were the host of focus. Consideration of the distribution of the population into either urban or rural settings will add deeper insights for future analysis, as this should include factors such as, but not limited to, malaria-related program, control, and research (such as the case for Bangladesh), environmental profile, meteorological conditions, presence of animal hosts and other potential vectors.

In addition to the difference in the geographical distribution between the two *P. ovale* spp., the higher proportion of observed *P. ovale curtisi* malaria cases than that of *P. ovale wallikeri* malaria cases in the meta-analysis could be explained by the type of blood samples (dried blood spots or venous blood) used for DNA extraction, the gene target investigated, or the PCR method used to differentiate *P. ovale* species. In the subgroup analysis, the real-time PCR method appeared to detect a higher proportion of *P. ovale curtisi* malaria cases than the nested PCR method. The nested PCR analysis suggests a relatively high false-negative rate or a lower sensitivity for detecting *Plasmodium* species compared with the real-time PCR method^5^. As an example, the protocol of Calderaro et al.^30^ can be cited, which was slightly less sensitive than that of Bauffe et al.^23^. These false-negative data might be caused by the protocol of the real-time PCR that requires two separate PCR assays and that exhibited higher cycle threshold (Ct) values in the case of failed detection of *P. ovale wallikeri* malaria^40^. Alternatively, the DNA extracted from dried blood spots might contain a low quantity of parasites or small fragments of degraded DNA, which are more likely to be detected with real-time PCR primers compared with primers designed for nested PCR that detect larger target sizes^43^. Furthermore, the resolution of agarose gels in nested PCR is lower than that in fluorescence detection by real-time PCR^43^. Moreover, the identification of *P. ovale* spp. that depends on the characterization of *SSU rRNA* dimorphism can be compromised by mutations or genetic polymorphisms in the *SSU rRNA* gene^33^ and may result in incorrect identification of *P. ovale* species. Therefore, some *P. ovale* infections mixed with other *Plasmodium* species at a very low parasite density could be undiagnosed by the *SSU rRNA*-based PCR method^13^. The PCR amplification of *SSU rRNA* in combination with other potential gene targets may have contributed to the higher proportion of *P. ovale curtisi* malaria cases identified in the included studies.

The potential explanations for the lower proportion of *P. ovale wallikeri* than *P. ovale curtisi* malaria cases are a low entomological inoculation rate (EIR), low relapse frequency, and the absence of dormancy of *P. ovale wallikeri*^43^. One potential difference between *P. ovale curtisi* and *P. ovale wallikeri* is the host erythrocyte preference, such as blood groups, which can restrict the two parasites to separate human populations^26^. However, mixed infections of both *P. ovale* spp*.* have been reported in studies conducted in Gabon and Bangladesh^41,43^. Hence, the blood group hypothesis, at present, does not support the difference between *P. ovale curtisi* and *P. ovale wallikeri*. The two *P. ovale* spp. may have distinguishing clinical characteristics such as the latency period. In the present meta-analysis, it was observed that the mean latency period of *P. ovale curtisi* was longer than that of *P. ovale wallikeri* malaria. This result was detected with high heterogeneity (94%) across the included studies, as the mean latency period of *P. ovale curtisi* was longer than that of *P. ovale wallikeri* in the four included studies conducted in Spain and China^47,48,52,53^, whereas the mean latency period of *P. ovale* spp. showed no difference in three other studies^30,32,40^, and the highest difference in the mean latency period of the two species was demonstrated in the study conducted by Rojo-Marcos et al.^48^. In the study conducted by Nolder et al., the longest recorded period of latency was 1083 days for *P. ovale curtisi* infection, and the mean latency period of *P. ovale curtisi* was shorter than that of *P. ovale wallikeri*^34^. This shows that *P. ovale curtisi* is probably more frequently asymptomatic, and the long latency period of *P. ovale curtisi* malaria makes it quite difficult to diagnose imported malaria cases in non-endemic countries, as the hypnozoites exist in the liver for months or years after infection without clinical illness^1^, although this hypothesis was not unequivocally proven. If the hypnozoites exist in the liver without specific treatment with primaquine for complete clearance, there could be a relapse of *P. ovale* spp. malaria, which might, with low evidence, lead to severe malaria^2^.

The incidence of imported malaria in non-endemic countries has been increasing due to the increase in international travel and migration. Travelers who visit malaria-endemic countries are suggested to take chemoprophylaxis against malaria infection. However, evidence suggests that exposing travelers to chemoprophylaxis could not resolve *P. ovale* spp. malaria infection but could be effective with other *Plasmodium* species^64^. Chemoprophylaxis is deemed ineffective against hypnozoites because it does not act in the liver, resulting in a long latency period of *P. ovale* infection before developing signs and symptoms^47^. Further studies on the latency period of these two species are required to improve the diagnosis and management of imported *P. ovale* spp. malaria.

The two *P. ovale* spp. may have distinguishing demographic characteristics such as age and sex. A previous study demonstrated that *P. ovale wallikeri* predominantly infected men and Caucasian subjects compared with *P. ovale curtisi*^47^. Moreover, the present meta-analysis of age using six studies^13,34,40,41,47,48^ with 133 *P. ovale* spp. malaria cases demonstrated no significant difference in age. Similar to age characteristics, there was no significant difference in gender between the two species, indicating that there is no selection of the host population during the infections of the two species. This study also investigated the differences in laboratory parameters, including parasite density, hemoglobin level, total leukocyte count, and platelet count. A previous study reported that parasite density significantly affected hematological parameters, particularly hemoglobin level, leukocyte count, and platelet count^65^. The results of the present study demonstrated no significant difference in parasite density and hemoglobin level, although a higher parasite density of *P. ovale curtisi* was reported in two studies conducted by Rojo-Marcos et al.^47,48^.

Nevertheless, the meta-analysis of those two studies^47,48^ demonstrated a higher leukocyte count in *P. ovale curtisi* infection than in *P. ovale wallikeri* infection. A previous study reported a reduction in total leukocyte counts during malaria infection^66^. In the present study, although the results demonstrated the difference in the mean leukocyte counts between *P. ovale curtisi* and *P. ovale wallikeri* malaria, the interpretation could not be made because only a small number of cases were investigated, or the difference suggested that *P. ovale curtisi* infection induces a higher immune response in patients than *P. ovale wallikeri* infection. A low platelet count during malaria infection is common^67^, but its pathogenesis is not completely understood. The results of the present meta-analysis demonstrated a lower platelet count in *P. ovale wallikeri* infection than in *P. ovale curtisi* infection. Patients with *P. ovale wallikeri* infection had low platelet counts or thrombocytopenia, and the rationale for this thrombocytopenia in *P. ovale wallikeri* infection but not in *P. ovale curtisi* infection remains unknown in the present study. Further cohort studies with a large sample of *P. ovale* spp. malaria cases are required. Although thrombocytopenia or severe thrombocytopenia is not included in the current WHO criteria for defining severe *P. ovale* spp. malaria^68^, it can be used as an indicator of malaria severity^40^ and predictor of mortality^69^.

There were several limitations in the present study. First, the prevalence of *P. ovale* spp. in some countries was derived from a small sample of research studies that are likely to be an underestimation of the total number of cases of each of *P. ovale* spp., or some countries do not routinely test/report *P. ovale* spp. cases. The underestimation of the number of *P. ovale* spp. cases were because of the diagnosis of *P. ovale* spp. malaria in endemic countries was dependent on the microscopic method that cannot differentiate the two distinct *P. ovale* spp. Second, most of the analysis was performed with a small subset of fewer than 200 cases per *P. ovale* spp., and the majority of *P. ovale* spp. were imported cases. Therefore, the results should be interpreted carefully. Third, the data on geographical mapping do not completely represent the *P. ovale* spp. cases in countries/continents but are data from reports from the studied populations in the respective regions. Fourth, other factors that could affect the differences in the clinical features of *P. ovale* spp., such as concurrent infection with other malaria parasite species, clinical versus asymptomatic presentation, age, malnutrition, and immune status, were not analyzed in this study. Fifth, the treatment data for *P. ovale wallikeri* and *P. ovale curtisi* malaria cases were limited and could not be analyzed. Sixth, regarding the calculation of the score, the data used in this study were research data from a probably limited group of researchers with particular awareness on *P. ovale* spp. malaria and not countrywide surveillance data and are, therefore, likely biased. Seventh, case reports were excluded in the present study as they do not contain prevalence data (the primary outcome). Eighth, the *P. ovale* cases included in the meta-analysis are probably a large underrepresentation of the number of cases per country, and the majority of the data was derived from imported cases, thereby indicating that it is not a real estimation of the frequency in the countries. Therefore, caution is necessary when interpreting the results of this meta-analysis.

In conclusion, the present study has demonstrated that malaria caused by *P. ovale curtisi* consisted of higher proportions of imported cases and had longer latency periods, higher mean platelet counts and higher mean total leukocyte counts than malaria caused by *P. ovale wallikeri*. Further studies are required to confirm the differences or similarities between these two species to promote a deeper understanding in terms of parasite biology and enhance malaria eradication programs.

## Supplementary Information

Supplementary Legends.

Supplementary Figure 1.

Supplementary Figure 2.

Supplementary Figure 3.

Supplementary Table S1.

Supplementary Table S2.

Supplementary Table S3.

Supplementary Table S4.
