# Supplementary material for: Comparison of Plasmodium ovale curtisi and Plasmodium ovale wallikeri infections by a meta-analysis approach
Source: Sci Rep. 2021 Mar 19;11:6409. doi: 10.1038/s41598-021-85398-w (PMC7979700; doi:10.1038/s41598-021-85398-w)
Supplement: Supplementary file 1 — Supplementary Legends. [file 41598_2021_85398_MOESM1_ESM.docx]

**Supplementary files**

**Supplementary figure 1.** Subgroup analysis of different PCR methods for identifying *P. ovale* spp.

The proportion of patients with *P. ovale curtisi* malaria compared to that of patients with *P. ovale wallikeri* malaria by different PCR methods. IV: Inverse variance, CI: Confidence interval, Event: Number of patients with *P. ovale curtisi* or *P. ovale wallikeri*, Random: Random-effects model, Total: Number of all *P. ovale* spp. cases, Lower in *curtisi*: The proportion of patients with *P. ovale curtisi* malaria was lower than that of patients with *P. ovale wallikeri* malaria. Higher in *curtisi*: The proportion of patients with *P. ovale curtisi* malaria was higher than that of patients with *P. ovale wallikeri* malaria.

**Supplementary figure 2.** Subgroup analysis of different target genes for PCR methods for identifying *P. ovale* spp.

The proportion of patients with *P. ovale curtisi* malaria compared to that of patients with *P. ovale wallikeri* malaria by different target genes for PCR methods. IV: Inverse variance, CI: Confidence interval, Event: Number of patients with *P. ovale curtisi* or *P. ovale wallikeri*, Random: Random-effects model, Total: Number of all *P. ovale* spp. cases, Lower in *curtisi*: The proportion of patients with *P. ovale curtisi* malaria was lower than that of patients with *P. ovale wallikeri* malaria. Higher in *curtisi*: The proportion of patients with *P. ovale curtisi* malaria was higher than that of patients with *P. ovale wallikeri* malaria.

**Supplementary figure 3.** Subgroup analysis of different blood samples for DNA extraction

The proportion of patients with *P. ovale curtisi* malaria compared to that of patients with *P. ovale wallikeri* malaria by different blood samples for DNA extraction. IV: Inverse variance, CI: Confidence interval, Event: Number of patients with *P. ovale curtisi* or *P. ovale wallikeri*, Random: Random-effects model, Total: Number of all *P. ovale* spp. cases, Lower in *curtisi:* The proportion of patients with *P. ovale curtisi* malaria was lower than that of patients with *P. ovale wallikeri* malaria. Higher in *curtisi*: The proportion of patients with *P. ovale curtisi* malaria was higher than that of patients with *P. ovale wallikeri* malaria.

**Table S1.** Search terms

The search terms used for retrieving the potentially relevant studies.

**Table S2.** Characteristics of the included studies

Characteristics of all included studies reporting *P. ovale* spp..

**Table S3.** Quality of the included studies

Quality of the included studies assessed by NOS scale.

**Table S4.** *P. ovale* host (human) burden score

*P. ovale* host (human) burden score is the number of reported cases of *P. ovale* malaria per country when compared to the host (human) density (1 sq. km: number of individuals).
