# Supplementary figures and images for: Comparison of Plasmodium ovale curtisi and Plasmodium ovale wallikeri infections by a meta-analysis approach
Source: Sci Rep. 2021 Mar 19;11:6409. doi: 10.1038/s41598-021-85398-w (PMC7979700; doi:10.1038/s41598-021-85398-w)

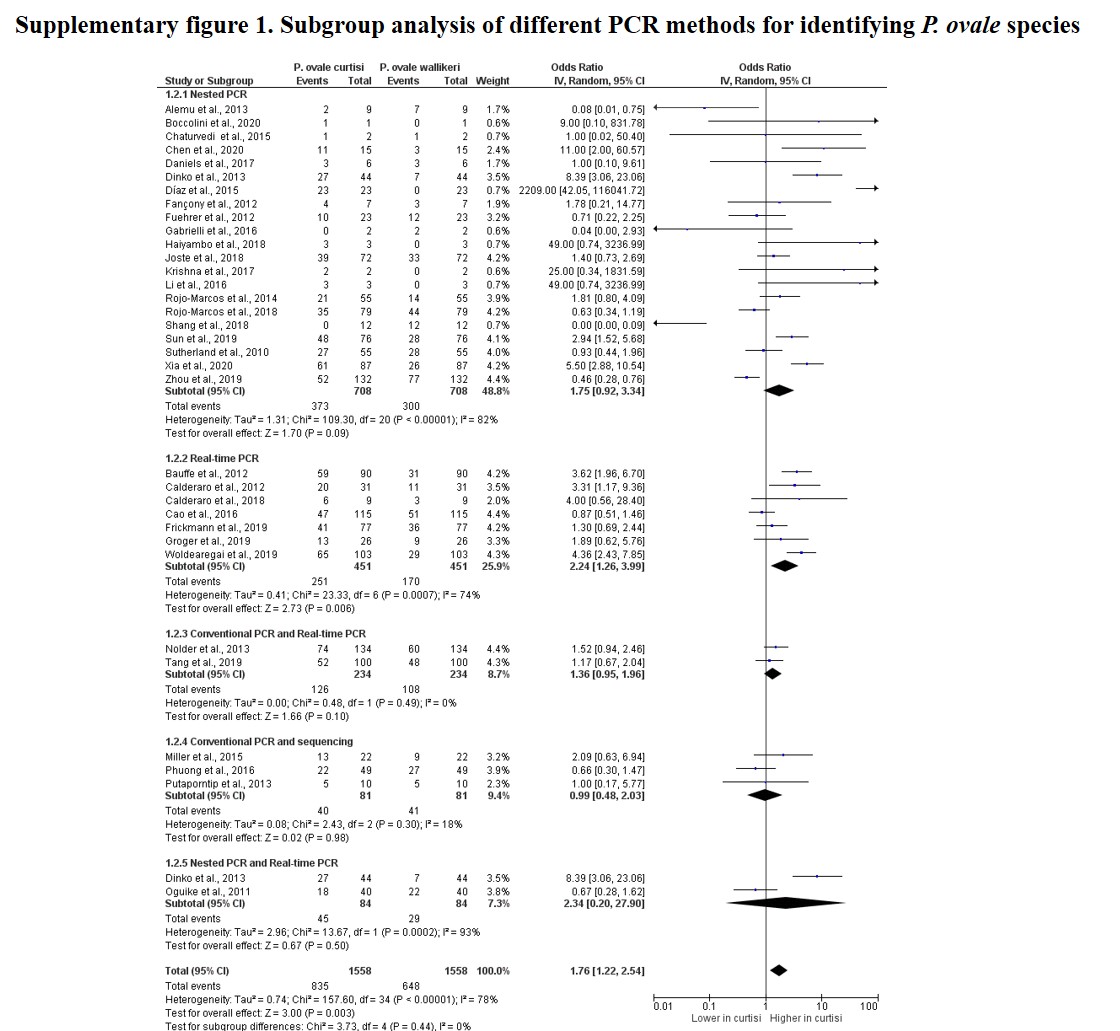

Supplement: Supplementary file 2 — Supplementary Figure 1. [file 41598_2021_85398_MOESM2_ESM.jpg]

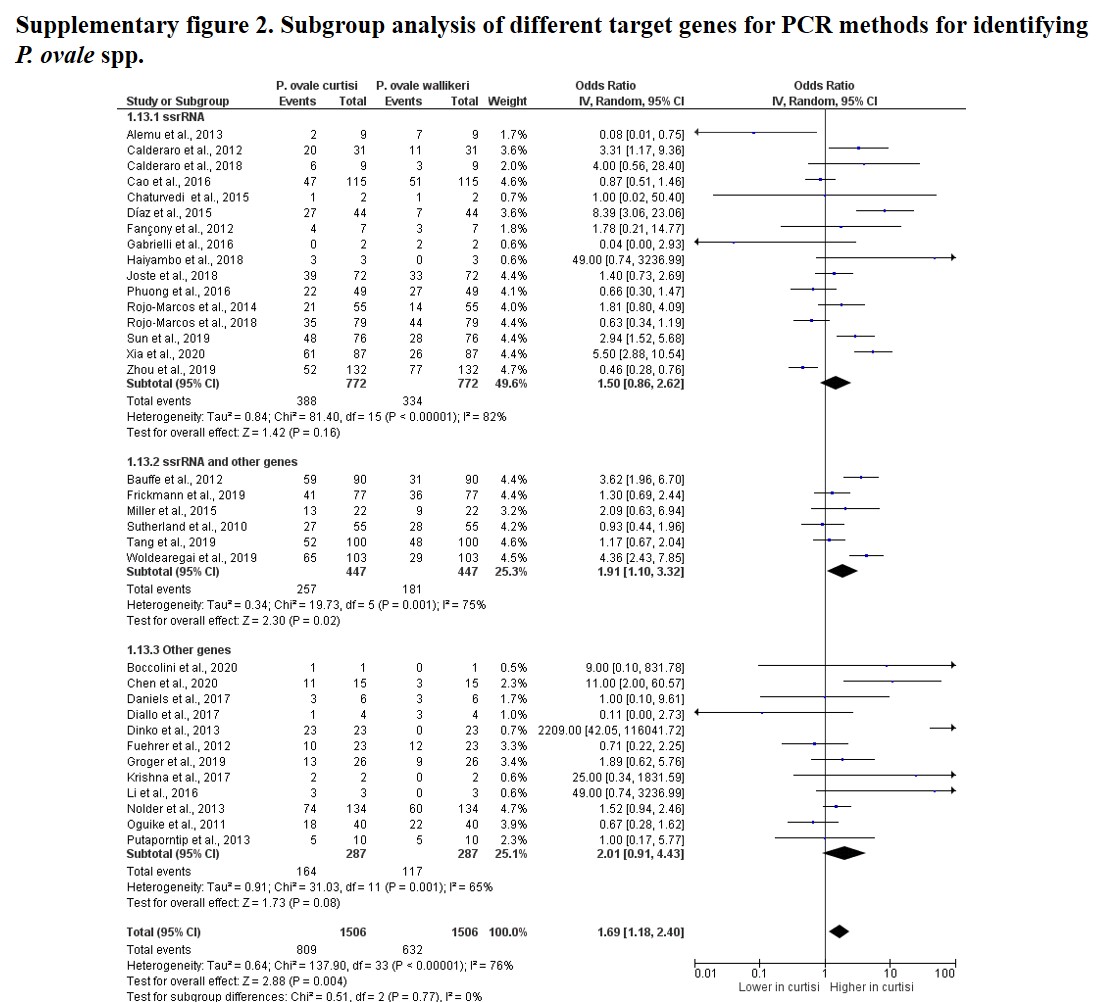

Supplement: Supplementary file 3 — Supplementary Figure 2. [file 41598_2021_85398_MOESM3_ESM.jpg]

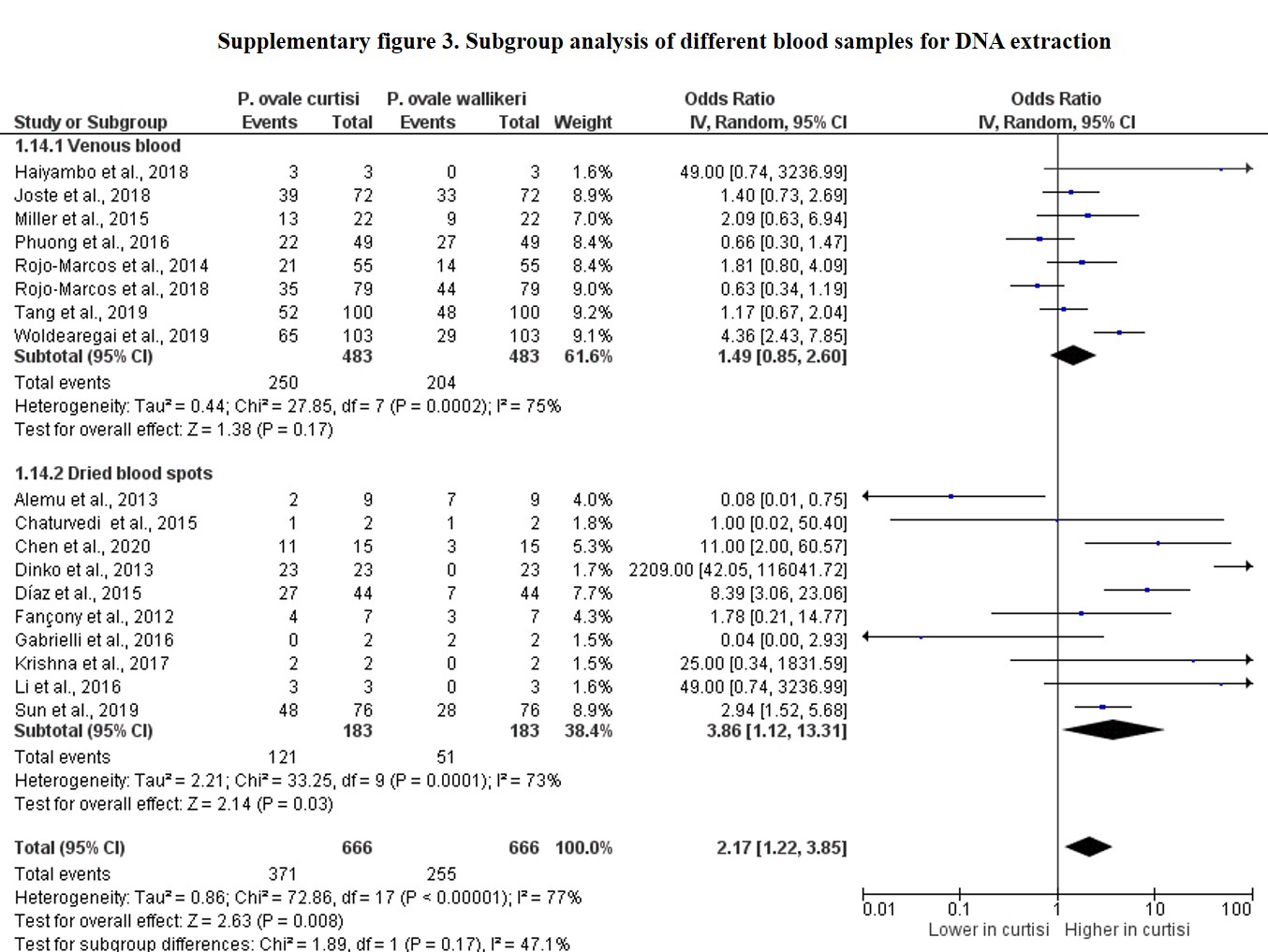

Supplement: Supplementary file 4 — Supplementary Figure 3. [file 41598_2021_85398_MOESM4_ESM.jpg]
