# Supplementary material for: Comparison of Plasmodium ovale curtisi and Plasmodium ovale wallikeri infections by a meta-analysis approach
Source: Sci Rep. 2021 Mar 19;11:6409. doi: 10.1038/s41598-021-85398-w (PMC7979700; doi:10.1038/s41598-021-85398-w)
Supplement: Supplementary file 5 — Supplementary Table S1. [file 41598_2021_85398_MOESM5_ESM.docx]

**Comparison of *Plasmodium ovale curtisi* and *Plasmodium ovale wallikeri* infections by a meta-analysis approach**

Aongart Mahittikorn^1^, Frederick Ramirez Masangkay^2^, Kwuntida Uthaisar Kotepui ^3^, Giovanni De Jesus Milanez^2^, Manas Kotepui^3*^

^1^ Department of Protozoology, Faculty of Tropical Medicine, Mahidol University, Bangkok, Thailand

^2^ Department of Medical Technology, Institute of Arts and Sciences, Far Eastern University-Manila, Manila, Philippines

^3^ Medical Technology, School of Allied Health Sciences, Walailak University, Tha Sala, Nakhon Si Thammarat, Thailand

Authors’ Email Addresses:

**^*^Corresponding Author**: Manas Kotepui; manas.ko@wu.ac.th, manaskote@gmail.com

Aongart Mahittikorn; aongart.mah@mahidol.ac.th

Frederick Ramirez Masangkay; frederick_masangkay2002@yahoo.com

Kwuntida Uthaisar Kotepui; kwuntida.ut@wu.ac.th

Giovanni De Jesus Milanez; gmilanez@feu.edu.ph

**Table S1.** **Search terms**

The search terms used for retrieving the potentially relevant studies.

| **Databases** | **Search terms** | **Search date** |
| --- | --- | --- |
| MEDLINE | (Plasmodium OR malaria) AND ovale AND (variant OR dimorphism OR subspecies OR curtisi OR wallikeri) | 24 July 2020 |
| Scopus | (Plasmodium OR malaria) AND ovale AND (variant OR dimorphism OR subspecies OR curtisi OR wallikeri)  Search option: All fields | 24 July 2020 |
| ISI Web of Science | (Plasmodium OR malaria) AND ovale AND (variant OR dimorphism OR subspecies OR curtisi OR wallikeri)  Search option: All fields | 24 July 2020 |
