# Supplementary material for: Comparison of Plasmodium ovale curtisi and Plasmodium ovale wallikeri infections by a meta-analysis approach
Source: Sci Rep. 2021 Mar 19;11:6409. doi: 10.1038/s41598-021-85398-w (PMC7979700; doi:10.1038/s41598-021-85398-w)
Supplement: Supplementary file 7 — Supplementary Table S3. [file 41598_2021_85398_MOESM7_ESM.docx]

**Comparison of *Plasmodium ovale curtisi* and *Plasmodium ovale wallikeri* infections by a meta-analysis approach**

Aongart Mahittikorn^1^, Frederick Ramirez Masangkay^2^, Kwuntida Uthaisar Kotepui ^3^, Giovanni De Jesus Milanez^2^, Manas Kotepui^3*^

^1^ Department of Protozoology, Faculty of Tropical Medicine, Mahidol University, Bangkok, Thailand

^2^ Department of Medical Technology, Institute of Arts and Sciences, Far Eastern University-Manila, Manila, Philippines

^3^ Medical Technology, School of Allied Health Sciences, Walailak University, Tha Sala, Nakhon Si Thammarat, Thailand

Authors’ Email Addresses:

**^*^Corresponding Author**: Manas Kotepui; manas.ko@wu.ac.th, manaskote@gmail.com

Aongart Mahittikorn; aongart.mah@mahidol.ac.th

Frederick Ramirez Masangkay; frederick_masangkay2002@yahoo.com

Kwuntida Uthaisar Kotepui; kwuntida.ut@wu.ac.th

Giovanni De Jesus Milanez; gmilanez@feu.edu.ph

**Table S3.** Quality of the included studies.

Quality of the included studies assessed by NOS scale.

| **No.** | **Reference** | **Selection** | | | | **Compatibility** | **Exposure** | | | **Total score (5)** | **Rating (High, moderate, low quality)** |
| --- | --- | --- | --- | --- | --- | --- | --- | --- | --- | --- | --- |
|  |  | **Is the Case Definition Adequate?** | **Representativeness of the Cases** | **Selection of Controls** | **Definition of Controls** |  | **Ascertainment of Exposure** | **Same method of ascertainment for cases and controls** | **Non – Response Rate** |  |  |
| 1. | Alemu et al., 2013 | **🟑** | **🟑** | NA | NA | **🟑🟑** | **🟑** | NA | NA | 5 | High |
| 2 | Bauffe et al., 2012 | **🟑** |  | NA | NA | **🟑🟑** | **🟑** | NA | NA | 4 | High |
| 3. | Boccolini et al., 2020 | **🟑** |  | NA | NA | **🟑🟑** | **🟑** | NA | NA | 4 | High |
| 4. | Calderaro et al., 2012 | **🟑** |  | NA | NA | **🟑🟑** | **🟑** | NA | NA | 4 | High |
| 5. | Calderaro et al., 2018 | **🟑** | **🟑** | NA | NA | **🟑🟑** | **🟑** | NA | NA | 5 | High |
| 6. | Cao et al., 2016 | **🟑** | **🟑** | NA | NA | **🟑🟑** | **🟑** | NA | NA | 5 | High |
| 7. | Chaturvedi et al., 2015 | **🟑** |  | NA | NA | **🟑🟑** | **🟑** | NA | NA | 4 | High |
| 8. | Chen et al., 2020 | **🟑** |  | NA | NA | **🟑🟑** | **🟑** | NA | NA | 4 | High |
| 9. | Chu et al., 2018 | **🟑** |  | NA | NA | **🟑🟑** | **🟑** | NA | NA | 4 | High |
| 10. | Daniels et al., 2017 | **🟑** | **🟑** | NA | NA | **🟑🟑** | **🟑** | NA | NA | 5 | High |
| 11. | Diallo et al., 2017 | **🟑** | **🟑** | NA | NA | **🟑🟑** | **🟑** | NA | NA | 5 | High |
| 12. | Díaz et al., 2015 | **🟑** | **🟑** | NA | NA | **🟑🟑** | **🟑** | NA | NA | 5 | High |
| 13. | Dinko et al., 2013 | **🟑** | **🟑** | NA | NA | **🟑🟑** | **🟑** | NA | NA | 5 | High |
| 14. | Fançony et al., 2012 | **🟑** | **🟑** | NA | NA | **🟑🟑** | **🟑** | NA | NA | 5 | High |
| 15. | Frickmann et al., 2019 | **🟑** |  | NA | NA | **🟑🟑** | **🟑** | NA | NA | 4 | High |
| 16. | Fuehrer et al., 2012 | **🟑** | **🟑** | NA | NA | **🟑🟑** | **🟑** | NA | NA | 5 | High |
| 17. | Gabrielli et al., 2016 | **🟑** | **🟑** | NA | NA | **🟑🟑** | **🟑** | NA | NA | 5 | High |
| 18. | Groger et al., 2019 | **🟑** |  | NA | NA | **🟑🟑** | **🟑** | NA | NA | 4 | High |
| 19. | Haiyambo et al., 2018 | **🟑** | **🟑** | NA | NA | **🟑🟑** | **🟑** | NA | NA | 5 | High |
| 20. | Joste et al., 2018 | **🟑** | **🟑** | NA | NA | **🟑🟑** | **🟑** | NA | NA | 5 | High |
| 21. | Krishna et al., 2017 | **🟑** | **🟑** | NA | NA | **🟑🟑** | **🟑** | NA | NA | 5 | High |
| 22. | Li et al., 2016 | **🟑** | **🟑** | NA | NA | **🟑🟑** | **🟑** | NA | NA | 5 | High |
| 23 | Miller et al., 2015 | **🟑** |  | NA | NA | **🟑🟑** | **🟑** | NA | NA | 4 | High |
| 24 | Nolder et al., 2013 | **🟑** |  | NA | NA | **🟑🟑** | **🟑** | NA | NA | 4 | High |
| 25 | Oguike et al., 2011 | **🟑** |  | NA | NA | **🟑🟑** | **🟑** | NA | NA | 4 | High |
| 26 | Phuong et al., 2016 | **🟑** |  | NA | NA | **🟑🟑** | **🟑** | NA | NA | 4 | High |
| 27 | Putaporntip et al., 2013 | **🟑** |  | NA | NA | **🟑🟑** | **🟑** | NA | NA | 4 | High |
| 28 | Rojo‑Marcos et al., 2018 | **🟑** |  | NA | NA | **🟑🟑** | **🟑** | NA | NA | 4 | High |
| 29 | Rojo-Marcos et al., 2014 | **🟑** |  | NA | NA | **🟑🟑** | **🟑** | NA | NA | 4 | High |
| 30 | Shang et al., 2018 | **🟑** |  | NA | NA | **🟑🟑** | **🟑** | NA | NA | 4 | High |
| 31 | Sun et al., 2019 | **🟑** | **🟑** | NA | NA | **🟑🟑** | **🟑** | NA | NA | 5 | High |
| 32 | Sutherland et al., 2010 | **🟑** |  | NA | NA | **🟑🟑** | **🟑** | NA | NA | 4 | High |
| 33 | Tang et al., 2019 | **🟑** |  | NA | NA | **🟑🟑** | **🟑** | NA | NA | 4 | High |
| 34 | Woldearegai et al., 2019 | **🟑** | **🟑** | NA | NA | **🟑🟑** | **🟑** | NA | NA | 5 | High |
| 35 | Xia et al., 2020 | **🟑** |  | NA | NA | **🟑🟑** | **🟑** | NA | NA | 4 | High |
| 36 | Zhou et al., 2019 | **🟑** | **🟑** | NA | NA | **🟑🟑** | **🟑** | NA | NA | 5 | High |

**🟑** A star system developed by the Newcastle-Ottawa Scale (NOS) for assessing the quality of non-randomized studies in meta-analyses, which consists of three domains including selection, comparability, and outcomes. A maximum of five stars (the highest quality) for the cross-sectional and retrospective studies was modified from the NOS for assessing the quality of the included studies. Any study rated ≥ 4 stars was considered a high-quality study.
